# Alcohol Use Disorder Diagnoses Among Individuals Who Take HIV Preexposure Prophylaxis

**DOI:** 10.1001/jamanetworkopen.2025.7295

**Published:** 2025-04-25

**Authors:** Anton L. V. Avanceña, Godwin Okoye, Rishit Yokananth, Aliza Norwood, Phillip W. Schnarrs

**Affiliations:** 1 Health Outcomes Division, College of Pharmacy, The University of Texas at Austin; 2 Department of Internal Medicine, Dell Medical School, The University of Texas at Austin; 3 Texas Institute for Sexual and Gender Minority Health Research, The University of Texas at Austin; 4 Department of Neuroscience, College of Natural Sciences, The University of Texas at Austin; 5 Department of Population Health, Dell Medical School, The University of Texas at Austin; 6 Now with: The Center for LGBT Health Research, Department of Behavioral and Community Health Sciences, School of Public Health, University of Pittsburgh, Pittsburgh, Pennsylvania

## Abstract

**Importance:**

Alcohol use disorder (AUD) may negatively affect preexposure prophylaxis (PrEP) adherence and continuation, reducing PrEP effectiveness.

**Objective:**

To estimate the prevalence of and and factors associated with AUD diagnoses among commercially insured individuals who take PrEP.

**Design, Setting, and Participants:**

This retrospective cohort study used US health care claims data to identify individuals aged 16 to 64 years who received at least 1 new PrEP prescription between January 1, 2014, to December 31, 2021. Data were analyzed from June 2024 to February 2025.

**Exposure:**

Sociodemographic characteristics included patient age, sex, geographic location, employment status, and type of health insurance. Clinical characteristics included sexually transmitted infection (STI) diagnosis and testing, use of psychotherapy services, and diagnosis of other mental health conditions.

**Main Outcomes and Measures:**

The main outcome was an AUD diagnosis within 6 months before or after the date of PrEP initiation. Among individuals with an AUD diagnosis, receipt of medications for AUD (MAUDs), including Food and Drug Administration (FDA)–approved MAUDs (acamprosate, disulfiram, and oral and injectable naltrexone) and non–FDA-approved MAUDs (baclofen, gabapentin, and topiramate) was determined.

**Results:**

The study cohort included 43 913 individuals receiving PrEP (mean [SD] age, 35.8 [10.94] years; 35 027 [90.1%] male assigned at birth). There were 6274 individuals (14.29%) who had an AUD diagnosis, with 1245 (2.84%) and 5029 (11.45%) receiving their diagnosis before and after PrEP initiation, respectively. The sociodemographic and clinical factors that were associated with an AUD diagnosis were similar whether AUD was diagnosed before or after PrEP initiation, including male sex assigned at birth (before: adjusted odds ratio [aOR], aOR, 0.62; 95% CI, 0.52-0.73; after: aOR, 0.81; 95% CI, 0.73-0.90) and the presence of other mental health diagnoses such as depression (before: aOR, 3.26; 95% CI, 2.78-3.84; after: aOR, 3.17; 95% CI, 2.88-3.49), anxiety (before: aOR, 2.16; 95% CI, 1.83-2.55; after: aOR, 2.24; 95% CI, 2.04-2.46), and any substance use disorder (before: aOR, 14.54; 95% CI, 12.46-16.96; after: aOR, 13.09; 95% CI, 11.82-14.49). There were 531 individuals with AUD diagnosis (8.46%) who received an FDA-approved MAUD and 883 (14.07%) who had a claim for a non–FDA-approved MAUD.

**Conclusions and Relevance:**

This population-based cohort study found that nearly 15% of individuals who took PrEP had an AUD diagnosis within 6 months of PrEP initiation; individuals with an AUD diagnosis were more likely to have co-occurring mental health conditions, and less than 9% received any FDA-approved MAUD. These findings suggest that interventions are needed to improve AUD services among individuals who take PrEP.

## Key Points

**Question:**

What is the prevalence of alcohol use disorder (AUD) diagnoses among people who take HIV preexposure prophylaxis (PrEP) with commercial insurance in the US?

**Findings:**

In this cohort study of 43 913 individuals who take PrEP, 14.29% had an AUD diagnosis (2.84% before and 11.45% after PrEP initiation). Mood disorders and substance use disorders were associated with AUD diagnosis. Only 8.46% of individuals with an AUD diagnosis received a US Food and Drug Administration (FDA)–approved medication for AUD.

**Meaning:**

The findings of this study suggest there is a need to improve AUD services among individuals who take PrEP.

## Teaser

This cohort study analyzes the prevalence of alcohol use disorder (AUD) diagnoses among commercially insured individuals who take preexposure prophylaxis (PrEP) as well as clinical and demographic characteristics associated with AUD.

## Introduction

Preexposure prophylaxis (PrEP) for HIV is a key prevention intervention in *Ending the HIV Epidemic*, the US national HIV strategy.^1^ The effectiveness of PrEP is dependent on each step of the PrEP care continuum—from awareness, uptake, adherence, and continuation of PrEP—which may be affected by factors like alcohol use.^2,3^ For example, individuals who use or engage in unhealthy alcohol use are often aware of PrEP but may hold misconceptions about interactions between alcohol and PrEP that moderate their willingness to use it.^4^ In terms of PrEP adherence and continuation (ie, persistence), findings from studies have been mixed. While there is evidence associating unhealthy alcohol use with PrEP nonadherence,^4,5^ other studies have found no association, suggesting that individuals who use alcohol can benefit from PrEP.^6,7,8,9,10^

Research involving individuals who take PrEP has found that unhealthy alcohol use is common. In one study of 172 men who have sex with men who used PrEP, 54% and 44% reported unhealthy alcohol use (defined as having a score ≥4 on the Alcohol Use Disorders Identification Test-Consumption questionnaire) and heavy episodic drinking, respectively.^11^ However, the prevalence of clinical diagnoses of alcohol use disorder (AUD) among individuals who take PrEP is less understood, which is the first and necessary step in the AUD treatment pathway.^12^ It is also unknown whether individuals who are prescribed PrEP initiate evidence-based treatments, specifically medications for AUD (MAUDs), which are effective in promoting abstinence and reducing unhealthy alcohol use.^13^

To build on this literature, we used a large, multiyear clinical dataset to estimate the prevalence of AUD diagnoses before and after PrEP initiation and to identify potential sociodemographic and clinical factors associated with AUD diagnoses. Among individuals who received an AUD diagnosis, we also sought to determine initiation of US Food and Drug Administration (FDA)–approved and selected non–FDA-approved MAUDs. Findings from this study can identify individuals who use PrEP whose alcohol use may interfere with PrEP adherence and continuation. Our results can also better characterize the intersection of AUD and HIV risk to inform health care practice and policy.

## Methods

### Study Design and Data Source

This retrospective cohort study follows the Strengthening the Reporting of Observational Studies in Epidemiology (STROBE) reporting guideline.^14^ We used Merative MarketScan data, which contains a convenience sample of more than 25 million individuals per year with employer-sponsored private health insurance and their dependents. The data include demographic information, diagnoses using *International Classification of Diseases, Ninth Revision (ICD-9) *and *International Statistical Classification of Diseases and Related Health Problems, Tenth Revision (ICD-10)* codes, and records for inpatient and outpatient services and outpatient prescriptions. Because MarketScan data are deidentified, this study was exempted for full review and the requirement of informed consent by The University of Texas Institutional Review Board.

### Study Population

We included individuals aged 16 to 64 years at the time of PrEP initiation from January 1, 2014, to December 31, 2021. The lower limit of the age range was informed by PrEP safety data from trials (available for adolescents aged ≥15 years) and by state laws that restrict autonomous consent among minors (generally <16 years).^15^ We restricted our age range to younger than 65 years because MarketScan includes primarily working-age adults and dependents. PrEP initiation was determined through an algorithm adopted in previous studies involving clinical data.^16^ First, we identified pharmacy claims for FDA-approved PrEP medications: 2 oral combination medications—tenofovir disoproxil fumarate/emtricitabine (TDF/FTC) and tenofovir alafenamide/emtricitabine (TAF/FTC)—and a long-acting injectable (cabotegravir). These medications were identified using National Drug Codes for the formulations used in PrEP (eTable 1 in Supplement 1). Second, we excluded patients who received a diagnosis of HIV or hepatitis B virus, which are indications for TDF/FTC, in the 6 months before their PrEP initiation (ie, index) date (eTable 2 in Supplement 1). We further limited the sample to individuals who had 6 months of continuous insurance coverage after their index date. This restriction, common in studies using administrative and electronic health record data, ensures that individuals in the sample have similar and sufficient observation periods.^17,18^

### Measures

#### Outcome

Our main outcome was an AUD diagnosis, which we identified using *ICD-10* codes in inpatient and outpatient services claims (eTable 3 in Supplement 1). We divided our sample into 3 groups based on the presence and timing of their AUD diagnosis. The AUD before initiation group included individuals with an AUD diagnosis in the baseline period or 6 months before PrEP initiation. A 6-month baseline period was selected because it aligns with the recommended duration of most AUD pharmacotherapies and psychotherapies.^19,20,21^ Among individuals without AUD diagnoses in the baseline period, we identified the AUD after initiation group, which includes those who received an AUD diagnosis in the follow-up period (ie, 6 months after PrEP initiation). Because individuals who use PrEP are advised to visit their PrEP clinicians every 3 months for HIV and sexually transmitted infection (STI) screening and laboratory testing,^22^ the 6-month follow-up period allowed us to capture AUD diagnoses within the first 2 routine visits after PrEP initiation. The final group included individuals without AUD diagnoses in the baseline or follow-up periods (no AUD).

Among individuals with an AUD diagnosis, we identified those who received an FDA-approved MAUD, including oral and injectable naltrexone, acamprosate, and disulfiram, after their AUD diagnosis. We also estimated receipt of non–FDA-approved MAUDs (baclofen, gabapentin, and topiramate), which are effective in treating AUD and are recommended as first- or second-line pharmacotherapy.^20,23,24^ Because health care payers do not always reimburse claims for medications used off-label,^25^ we could not ascertain whether non–FDA-approved MAUDs were prescribed for AUD or other indications.

#### Exposure Variables

Sociodemographic characteristics, including age, sex assigned at birth, insurance plan, employment status, and geographic region, were measured on the index date. We also included year of PrEP initiation as an exposure variable, which we grouped into 3 periods: 2014 to 2016 for the early years of PrEP uptake, 2017 to 2019 for expanded access to PrEP, and 2020 to 2021 for the height of the COVID-19 pandemic. We also captured several clinical characteristics during the 6-month baseline period, including diagnoses of depression, anxiety, posttraumatic stress disorder (PTSD), bipolar disorder, substance use disorders (SUDs) related to use of opioids (including opioid overdoses), nicotine, cannabis, stimulants, sedatives, and other substances using *ICD-9* and *ICD-10* codes that have been associated with PrEP nonadherence in prior studies (eTable 4 and eTable 5 in Supplement 1).^26,27^ We also assessed diagnoses of STIs at baseline, as well as use of STI screening and psychotherapy services (eTable 6 and eTable 7 in Supplement 1).

### Statistical Analysis

We used descriptive statistics to summarize baseline characteristics for individuals with and without AUD diagnosis. Statistical differences between the 2 groups were evaluated using the *t* test for continuous variables, Pearson χ^2^ test for binary variables, and Mantel-Haenszel χ^2^ test for nominal variables. We fitted a multivariable logistic regression model to determine the sociodemographic and clinical characteristics that were associated with AUD diagnosis before and after PrEP initiation. Missing data were excluded from the outcome analysis. We considered statistical significance to be a 2-sided *P* < .05. Analyses were conducted from June 2024 to February 2025 using SAS version 9.4 (SAS Institute).

## Results

Out of 132 737 individuals who had PrEP medication claims, 43 913 met the inclusion criteria. Most exclusions were due to insufficient continuous insurance enrollment around the date of PrEP initiation or after (eFigure in Supplement 1). The final sample had a mean (SD) age of 35.80 (10.94) years, 35 027 (90.10%) were male assigned at birth, 38 050 (86.65%) received TDF/FTC, and 5863 (13.35%) received TAF/FTC (eFigure and eTable 8 in Supplement 1). Most individuals initiated PrEP in 2017 to 2019 (19 330 individuals [44.02%]), followed by 2014 to 2016 (13 008 individuals [29.62%]) and 2020 to 2021 (11 575 individuals [26.36%]). There were 37 639 individuals who took PrEP without an AUD diagnosis (no AUD group).

### Prevalence of AUD Diagnoses

A total of 6274 individuals who took PrEP (14.29%) had an AUD diagnosis within 6 months of their PrEP initiation (Table 1). Of these individuals, 1245 (2.84%) and 5029 (11.45%), respectively, received an AUD diagnosis at baseline (AUD before initiation) and after initiating PrEP (AUD after initiation).

**Table 1.** Baseline Demographic and Clinical Characteristics of 43 913 Individuals Who Take PrEP by AUD Diagnosis (2014-2021)^a^

Characteristic	Individuals, No. (%)			*P* value	
	AUD before initiation (n = 1245)	AUD after initiation (n = 5029)	No AUD diagnoses (n = 37 639)	AUD before initiation vs no AUD	AUD after initiation vs no AUD
Age, mean (SD), y	34.88 (11.43)	35.49 (11.96)	35.85 (10.78)	<.001	.03
Sex at birth					
Male	1037 (83.29)	4345 (86.40)	33 990 (90.31)	<.001	<.001
Female	208 (16.71)	684 (13.60)	3649 (9.69)		
Index year					
2014-2016	391 (31.40)	1539 (30.60)	11 078 (29.43)	.16	<.001
2017-2019	544 (43.69)	2417 (48.06)	16 369 (43.49)		
2020-2021	310 (24.90)	1073 (21.34)	10 192 (27.08)		
Insurance plan					
HMO	176 (14.14)	773 (15.37)	5015 (13.32)	.33	<.001
POS	231 (18.55)	700 (13.92)	7539 (20.03)		
PPO	584 (46.91)	2407 (47.86)	16 987 (45.13)		
Others	254 (20.40)	1149 (22.85)	8098 (21.51)		
Employed	986 (79.20)	4109 (81.71)	31 409 (83.45)	<.001	<.001
Region					
Northeast	348 (27.95)	1342 (26.69)	9531 (25.32)	.07	<.001
Midwest	175 (14.06)	806 (16.03)	4833 (12.84)		
South	428 (34.38)	1758 (34.96)	13 897 (36.92)		
West	288 (23.13)	1112 (22.11)	9253 (24.58)		
Unknown	6 (0.48)	11 (0.22)	125 (0.33)		
Mental health comorbidities					
Depression	389 (31.24)	1357 (26.98)	1996 (5.30)	<.001	<.001
Anxiety	333 (26.75)	1186 (23.58)	2488 (6.61)	<.001	<.001
PTSD	94 (7.55)	317 (6.30)	738 (1.96)	<.001	<.001
Bipolar	162 (13.01)	547 (10.88)	590 (1.57)	<.001	<.001
Substance use disorders					
Opioids	65 (5.22)	259 (5.15)	90 (0.24)	<.001	<.001
Nicotine	164 (13.17)	497 (9.88)	476 (1.26)	<.001	<.001
Cannabis	121 (9.72)	538 (10.70)	116 (0.31)	<.001	<.001
Stimulants	100 (8.03)	298 (5.93)	103 (0.27)	<.001	<.001
Sedatives	64 (5.14)	204 (4.06)	29 (0.08)	<.001	<.001
Other	70 (5.62)	280 (5.57)	99 (0.26)	<.001	<.001
STI diagnosis	132 (10.60)	297 (5.91)	6700 (17.80)	<.001	<.001
STI screening	189 (15.18)	579 (11.51)	3806 (10.11)	<.001	<.001
Psychotherapy	173 (13.90)	535 (10.64)	2084 (5.54)	<.001	<.001

Abbreviations: AUD, alcohol use disorder; HMO, health maintenance organization; POS, point of service; PPO, preferred provider organization; PrEP, preexposure prophylaxis; PTSD, posttraumatic stress disorder; STI, sexually transmitted infection.

^a^ Comparisons were made between individuals with AUD diagnoses before or after initiation and individuals without AUD diagnoses. Statistical differences between the 2 groups were evaluated using the *t* test for continuous variables, Pearson χ^2^ test for binary variables, and Mantel-Haenszel χ^2^ test for nominal variables.

### Characteristics of AUD and No AUD Groups

Compared with the no AUD group, individuals in the AUD before initiation group were younger, less likely to be male assigned at birth, and less likely to be employed (Table 1). Individuals in the AUD before initiation group were also more likely to have prior diagnoses of depression, anxiety, PTSD, bipolar disorder, opioid use disorder, nicotine use disorder, cannabis use disorder, stimulant use disorder, sedative use disorder, and other SUDs.

The AUD after initiation and no AUD groups showed similar differences in baseline characteristics (Table 1). The AUD after initiation group was younger, less likely to be male assigned at birth, and less likely to be employed than the no AUD group. Individuals in the AUD after initiation group were also more likely to have prior diagnoses of depression, anxiety, PTSD, bipolar disorder, OUD, nicotine use disorder, cannabis use disorder, stimulant use disorder, sedative use disorder, and other SUDs.

Healthcare service utilization at baseline was also different between individuals with and without AUD diagnoses. Use of psychotherapy was more common in the AUD before initiation (173 individuals [13.9%] vs 2084 individuals [5.54%]; *P* < .001) and AUD after initiation (535 individuals [10.64%] vs 2084 individuals [5.54%]; *P* < .001) groups compared with the no AUD group. STI screening—but not STI diagnoses—was higher among individuals with an AUD diagnosis before or after PrEP initiation (Table 1).

### AUD Pharmacotherapy Treatment

Only 531 of 6274 individuals with an AUD diagnosis (8.46%) received an FDA-approved MAUD after their diagnosis. Among those in the AUD before initiation group (1245 individuals), most received oral naltrexone (146 individuals [11.73%]), followed by disulfiram (39 individuals [3.13%]) and equal numbers of acamprosate and injectable naltrexone recipients (8 individuals [0.64%]) (Figure).

**Figure.**
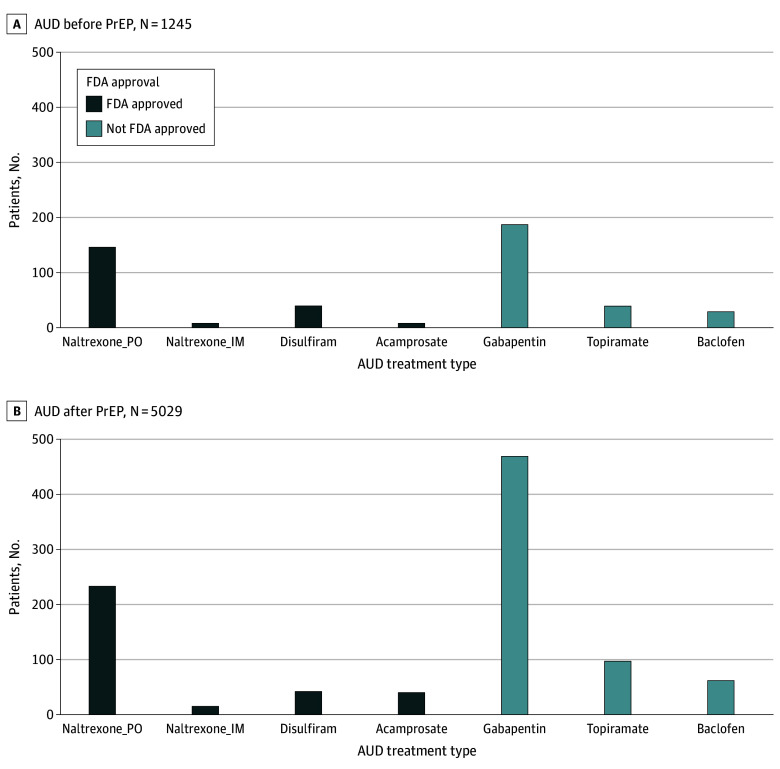
US Food and Drug Administration (FDA)–Approved Pharmacotherapy Treatment Among Individuals Who Take Preexposure Prophylaxis (PrEP) With Alcohol Use Disorder (AUD) Diagnosis IM indicates intramuscular administration; PO, oral administration.

Similar patterns were seen among the 5029 individuals in the AUD after initiation group; most received oral naltrexone (233 individuals [4.63%]), followed by disulfiram (42 individuals [0.84%]), acamprosate (40 individuals [0.80%]), and injectable naltrexone (15 individuals [0.30%]) (Figure).

Higher proportions of individuals in both AUD groups received non–FDA-approved MAUDs (Figure). There were 187 individuals in the AUD before initiation group (15.02%) and 469 individuals in the AUD after initiation group (9.33%) who had claims for gabapentin. Receipt of topiramate (39 individuals in the AUD before initiation group [3.13%] and 97 individuals in the AUD after initiation group [1.93%]) and baclofen (29 individuals in the AUD before initiation group [2.33%] and 62 individuals in the AUD after initiation group [1.23%]) were also more common than most FDA-approved MAUDs in the AUD before and after initiation groups.

### Factors Associated With AUD Diagnosis

There was a negative association of age (adjusted odds ratio [aOR], 0.99; 95% CI, 0.99-1.00; *P* = .01) and being male assigned at birth (aOR, 0.62; 95% CI, 0.52-0.73; *P* < .001) with an AUD diagnosis before PrEP initiation (Table 2). Diagnoses of depression (aOR, 3.26; 95% CI, 2.78-3.84; *P* < .001), anxiety (aOR, 2.16; 95% CI, 1.83-2.55; *P* < .001), PTSD (aOR, 2.09; 95% CI, 1.58-2.77; *P* < .001), bipolar disorder (aOR, 2.90; 95% CI, 2.30-3.67; *P* < .001), or any SUD (aOR, 14.54; 95% CI, 12.46-16.96; *P* < .001) were positively associated with an AUD diagnosis before PrEP initiation. We found that individuals residing in the Northeast (aOR, 1.25; 95% CI, 1.06-1.46; *P* = .01) and in the West (aOR, 1.19; 95% CI, 1.01-1.40; *P* = .04) were more likely to have an AUD diagnosis after PrEP initiation compared with the South.

**Table 2.** Results From Multivariable Logistic Regression Analysis^a^

Parameters	AUD before PrEP initiation		AUD after PrEP initiation	
	Adjusted odds ratio (95% CI)	*P* value	Adjusted odds ratio (95% CI)	*P* value
Age	0.99 (0.99-1.00)	.01	1.00 (1.00-1.00)	.73
Sex				
Female	1 [Reference]	NA	1 [Reference]	NA
Male	0.62 (0.52-0.73)	<.001	0.81 (0.73-0.90)	<.001
Index year				
2014-2016	1 [Reference]	NA	1 [Reference]	NA
2017-2019	1.12 (0.96-1.30)	.14	1.15 (1.07-1.25)	<.001
2020-2021	1.04 (0.88-1.24)	.65	0.84 (0.76-0.92)	<.001
Region				
South	1 [Reference]	NA	1 [Reference]	NA
Northeast	1.25 (1.06-1.46)	.01	1.15 (1.06-1.25)	.001
Midwest	1.13 (0.93-1.38)	.24	1.25 (1.13-1.39)	<.001
West	1.19 (1.01-1.40)	.04	1.02 (0.93-1.11)	.69
Depression diagnosis				
Without depression	1 [Reference]	NA	1 [Reference]	NA
With depression	3.26 (2.78-3.84)	<.001	3.17 (2.88-3.49)	<.001
Anxiety diagnosis				
Without anxiety	1 [Reference]	NA	1 [Reference]	NA
With anxiety	2.16 (1.83-2.55)	<.001	2.24 (2.04-2.46)	<.001
PTSD diagnosis				
Without PTSD	1 [Reference]	NA	1 [Reference]	NA
With PTSD	2.09 (1.58-2.77)	<.001	1.86 (1.57-2.21)	<.001
Bipolar disorder diagnosis				
Without bipolar disorder	1 [Reference]	NA	1 [Reference]	NA
With bipolar disorder	2.90 (2.30-3.67)	<.001	2.57 (2.21-2.99)	<.001
SUD				
Without SUD	1 [Reference]	NA	1 [Reference]	NA
With SUD^b^	14.54 (12.46-16.96)	<.001	13.09 (11.82-14.49)	<.001

Abbreviations: AUD, alcohol use disorder; NA, not applicable; PrEP, preexposure prophylaxis; PTSD, posttraumatic stress disorder; SUD, substance use disorder.

^a^ The results of the logistic regression analysis shown here are adjusted for age, sex, depression diagnoses, anxiety diagnoses, bipolar diagnoses, and nonalcohol SUDs.

^b^ SUDs include use of nicotine, cannabis, sedatives, stimulants (including cocaine and amphetamines), and other substances. These diagnoses were combined due to multicollinearity among these variables and model nonconvergence due to small numbers.

The factors associated with AUD diagnoses after PrEP initiation were similar (Table 2). We found that being male assigned at birth (aOR, 0.81; 95% CI, 0.73-0.90; *P* < .001), but not age, was negatively associated with an AUD diagnosis after PrEP initiation. Diagnoses of depression (aOR, 3.17; 95% CI, 2.88-3.49; *P* < .001), anxiety (aOR, 2.24; 95% CI, 2.04-2.46; *P* < .001), PTSD (aOR, 1.86; 95% CI, 1.57-2.21, *P* < .001), bipolar disorder (aOR, 2.57; 95% CI, 2.21-2.99; *P* < .001), and any SUD (aOR, 13.09; 95% CI, 11.82-14.49; *P* < .001) were associated with higher odds of an AUD diagnosis after PrEP initiation. We also found that selected regions of residence and years of PrEP initiation were associated with an AUD diagnosis.

## Discussion

This cohort study of 43 913 individuals who take PrEP found that nearly 15% had an AUD diagnosis within 6 months of initiating PrEP. The sociodemographic and clinical factors that were associated with an AUD diagnosis were similar whether AUD was diagnosed before or after PrEP initiation. Among individuals who received an AUD diagnosis, receipt of AUD treatments was low, with less than 9% of individuals receiving an FDA-approved MAUD.

The prevalence of AUD diagnoses we identified in this study was higher than the past-year AUD prevalence among US adults in 2023 (10.9%), which is based on self-reported symptoms and alcohol consumption.^28^ Our AUD diagnosis prevalence also differed from estimates in prior studies that involved people who use PrEP. For example, an analysis of 13 906 individuals who take PrEP from Kaiser Permanente^9^ reported that 25.2% had received an AUD diagnosis at initiation, which is significantly lower than our prevalence before PrEP initiation (2.84%). Given the underdiagnosis of AUD,^12^ it is possible that individuals in our study who received an AUD diagnosis after PrEP initiation (11.45%) were already experiencing AUD symptoms in the baseline period or earlier. Several studies that measured unhealthy alcohol use among individuals who take PrEP or who are at risk for HIV (eg, men who have sex with men) suggest that the burden of AUD is much higher than what we observed.^11,29,30^

PrEP guidelines recommend universal screening for AUD and other SUDs among adults who take PrEP^22^; unfortunately, the prevalence, timing, and quality of AUD screening in this population has not been studied. In the general population, several barriers have been associated with suboptimal AUD screening and diagnosis, including stigma associated with having an AUD diagnosis, belief that AUD will resolve on its own, and service-level factors such as lack of clinician knowledge and training about AUD and high clinical workloads.^31,32^ Future research should understand the specific AUD screening barriers experienced by individuals who take PrEP.

Several sociodemographic and clinical factors were associated with AUD diagnoses before and after PrEP initiation. Being male assigned at birth was associated with lower odds of AUD diagnosis, which does not reflect the higher burden of AUD and unhealthy alcohol use in this population compared with females (or men vs women).^28^ This association may be due to a prior finding that men are less likely to be asked about their drinking by clinicians, reducing the probability of receiving an AUD diagnosis.^33^ However, that same study found that when men are asked about their alcohol use, they are more likely to be asked about possible misuse, advised to reduce consumption, and offered treatment information compared with women.

We also found that several mental health diagnoses were associated with AUD, aligning with previous research showing the co-occurrence of AUD with these conditions.^34^ Individuals who take PrEP have also been found to use more substances, including alcohol, compared with peers who do not take PrEP.^35^ These results suggest that individuals who are taking PrEP and receive an AUD diagnosis may benefit from additional screenings and holistic mental health treatment, potentially through their PrEP clinician with whom they have routine visits. STI testing was more common among individuals with AUD, which may explain the lower proportion of STI diagnoses in this group compared with those without an AUD diagnosis.

Improvements in AUD treatment may mitigate alcohol-related harms, including suboptimal PrEP adherence and continuation. Our study found that only 8.46% of individuals who received an AUD diagnosis received FDA-approved MAUDs, which is higher than the 2.0% of US adults with AUD who received MAUDs.^28^ Our study’s higher AUD treatment prevalence may be due to 100% health care coverage in our sample. The higher treatment prevalence may also be due to the heightened trust individuals place in their PrEP clinicians^8^ and the increased frequency of clinician visits that users of PrEP are recommended to have, which offers more opportunities for SUD treatment.^36^ However, this population’s overall low prevalence of MAUD initiation requires further investigation. We also found that 14.07% of individuals in our study had claims for a non–FDA-approved MAUD—a prevalence that is 66% higher than FDA-approved MAUDs. While we cannot ascertain whether these medications were prescribed for AUD treatment specifically, our findings highlight an opportunity to examine the effects of these non–FDA-approved MAUDs.

Interventions are likely needed to address unhealthy alcohol use among users of PrEP and to increase AUD treatment rates. While brief and multisession psychosocial interventions for AUD are effective in the general population,^37,38^ their effectiveness with individuals who take PrEP is limited. Pilot trials of multisession, motivational interviewing-based interventions to reduce substance use have found modest or no effects on alcohol use among sexual and/or gender minority adolescents and young adults who are using PrEP.^39,40^ Null results were also reported in a trial of a web-based app that provided personalized feedback and text reminders to individuals taking PrEP.^41^ The lack of efficacy may be due to several factors, including the short duration of interventions, the lack of interest among participants to address unhealthy alcohol use and PrEP concurrently, and the syndemic challenges that are common among individuals who take PrEP (eg, socioeconomic insecurity) that can lead to dropout.^39,40,41^ One promising approach is targeted oral naltrexone use, or taking naltrexone pills before periods of anticipated heavy drinking, which offers a MAUD-based strategy to reduce unhealthy alcohol use among individuals taking PrEP and other individuals at risk for HIV.^42^

### Limitations

This study has limitations. By using health care claims data, we may have missed diagnoses or health care services received without insurance or outside traditional health care settings. For example, STI testing and treatment are often offered through community-based organizations that do not require health insurance. Adolescents and young adults whose health care coverage is through their primary caregivers may also choose to receive PrEP through community access programs that bypass their health insurance to avoid disclosure.^43,44^ MarketScan only includes individuals with employer-sponsored commercial insurance and leaves out other populations (eg, Medicaid beneficiaries or uninsured individuals); thus, the generalizability of our findings is limited. MarketScan also lacks information on key variables such as gender, sexual orientation, race, and ethnicity, which are important determinants of both AUD and PrEP initiation. For example, studies have documented significantly lower PrEP uptake among Black and Hispanic individuals, which likely contributes to growing ethnic and racial disparities in HIV.^45^ Our algorithm for identifying individuals who take PrEP in clinical data, while highly sensitive and specific^16^ and routinely used for PrEP surveillance in the US,^45^ may have missed or misclassified individuals. Imposing a minimum continuous enrollment criterion, which led to sizeable exclusions in our study, may have introduced selection bias. However, having a sufficient look-back period was necessary to ascertain PrEP initiation.^16^ Additionally, adjusting estimates derived from samples with enrollment restrictions, as this study did, may reduce or eliminate any biases.^17^ Furthermore, AUD is underdiagnosed and may have led to the underestimation of AUD among individuals in our sample.

## Conclusions

This cohort study found that nearly 15% of individuals who initiated PrEP had AUD diagnoses 6 months before or after PrEP initiation. Users of PrEP with an AUD diagnosis were more likely to have co-occurring mental health conditions, including other SUDs, and less than 10% received any FDA-approved MAUD. Interventions are needed to improve screening and treatment of AUD among individuals who take PrEP to reduce the risks associated with unhealthy alcohol use.

## Notes

Supplement 1. **eTable 1.** National Drug Codes for FDA-Approved PrEP Medications **eTable 2.** Diagnosis Codes for Viral Diseases Treated With TDF/FTC **eTable 3.** Diagnosis Codes for Alcohol Use Disorder **eTable 4.** Diagnosis Codes for Mental Health Conditions **eTable 5.** Diagnosis Codes for Substance Use Disorders **eTable 6.** Diagnosis and Service Codes for STI Diagnoses and Testing **eTable 7.** Diagnosis and Service Codes Used to Identify Psychosocial Therapy **eFigure.** CONSORT Diagram Showing the Cohort Selection of Individuals Who Use PrEP With and Without AUD Diagnoses **eTable 8.** Baseline Characteristics of Full Sample

Supplement 2.
Data Sharing Statement

## References

[1] Centers for Disease Control and Prevention. Estimated HIV incidence and prevalence in the United States, 2018-2022. Published May 21, 2024. Accessed March 18, 2025. https://stacks.cdc.gov/view/cdc/156513

[2] Kelley CF, Kahle E, Siegler A, . Applying a PrEP continuum of care for men who have sex with men in Atlanta, Georgia. Clin Infect Dis. 2015;61(10):1590-1597. doi:10.1093/cid/civ664 26270691 PMC4614414

[3] Nunn AS, Brinkley-Rubinstein L, Oldenburg CE, . Defining the HIV pre-exposure prophylaxis care continuum. AIDS. 2017;31(5):731-734. doi:10.1097/QAD.0000000000001385 28060019 PMC5333727

[4] Oldfield BJ, Edelman EJ. Addressing unhealthy alcohol use and the HIV pre-exposure prophylaxis care continuum in primary care: a scoping review. AIDS Behav. 2021;25(6):1777-1789. doi:10.1007/s10461-020-03107-6 33219492 PMC8084877

[5] Gebru NM, Canidate SS, Liu Y, . Substance use and adherence to HIV pre-exposure prophylaxis in studies enrolling men who have sex with men and transgender women: a systematic review. AIDS Behav. 2023;27(7):2131-2162. doi:10.1007/s10461-022-03948-3 36538138 PMC10869193

[6] Hoenigl M, Jain S, Moore D, ; California Collaborative Treatment Group 595 Team. Substance use and adherence to HIV preexposure prophylaxis for men who have sex with men. Emerg Infect Dis. 2018;24(12):2292-2302. doi:10.3201/eid2412.180400 30457536 PMC6256399

[7] Miller AP, Shoptaw S, Moucheraud C, . Recent alcohol use is associated with increased pre-exposure prophylaxis (PrEP) continuation and adherence among pregnant and postpartum women in South Africa. J Acquir Immune Defic Syndr. 2023;92(3):204-211. doi:10.1097/QAI.0000000000003133 36413977 PMC9928886

[8] Strong SH, Oldfield BJ, van den Berg JJ, . Perspectives on unhealthy alcohol use among men who have sex with men prescribed HIV pre-exposure prophylaxis: a qualitative study. Prev Med Rep. 2023;37:102553. doi:10.1016/j.pmedr.2023.102553 38282665 PMC10810836

[9] Hojilla JC, Hurley LB, Marcus JL, . Characterization of HIV preexposure prophylaxis use behaviors and HIV incidence among US adults in an integrated health care system. JAMA Netw Open. 2021;4(8):e2122692. doi:10.1001/jamanetworkopen.2021.22692 34436609 PMC8391097

[10] Watson RJ, Morgan E, Collibee C, . Substance use and healthcare utilization across the pre-exposure prophylaxis (PrEP) care cascade among Black and Latino men who have sex with men. Subst Use Misuse. 2022;57(11):1698-1707. doi:10.1080/10826084.2022.2108059 35938746 PMC9554788

[11] Ogbuagu O, Marshall BDL, Tiberio P, . Prevalence and correlates of unhealthy alcohol and drug use among men who have sex with men prescribed HIV pre-exposure prophylaxis in real-world clinical settings. AIDS Behav. 2019;23(1):190-200. doi:10.1007/s10461-018-2260-9 30145707 PMC7020905

[12] Mintz CM, Hartz SM, Fisher SL, . A cascade of care for alcohol use disorder: using 2015-2019 National Survey on Drug Use and Health data to identify gaps in past 12-month care. Alcohol Clin Exp Res. 2021;45(6):1276-1286. doi:10.1111/acer.14609 33993541 PMC8254783

[13] McPheeters M, O’Connor EA, Riley S, . Pharmacotherapy for alcohol use disorder: a systematic review and meta-analysis. JAMA. 2023;330(17):1653-1665. doi:10.1001/jama.2023.19761 37934220 PMC10630900

[14] von Elm E, Altman DG, Egger M, Pocock SJ, Gøtzsche PC, Vandenbroucke JP; STROBE Initiative. The Strengthening the Reporting of Observational Studies in Epidemiology (STROBE) statement: guidelines for reporting observational studies. PLoS Med. 2007;4(10):e296. doi:10.1371/journal.pmed.0040296 17941714 PMC2020495

[15] Tanner MR, Miele P, Carter W, . Preexposure prophylaxis for prevention of HIV acquisition among adolescents: clinical considerations, 2020. MMWR Recomm Rep. 2020;69(3):1-12. doi:10.15585/mmwr.rr6903a1 32324724 PMC7188407

[16] Furukawa NW, Smith DK, Gonzalez CJ, . Evaluation of algorithms used for PrEP surveillance using a reference population from New York City, July 2016–June 2018. Public Health Rep. 2020;135(2):202-210. doi:10.1177/0033354920904085 32027559 PMC7036610

[17] Shortreed SM, Johnson E, Rutter CM, Kamineni A, Wernli KJ, Chubak J. Cohort restriction based on prior enrollment: examining potential biases in estimating cancer and mortality risk. Obs Stud. 2016;2(1):51-64. doi:10.1353/obs.2016.0002 28530002 PMC5435370

[18] Jensen ET, Cook SF, Allen JK, . Enrollment factors and bias of disease prevalence estimates in administrative claims data. Ann Epidemiol. 2015;25(7):519-525.e2. doi:10.1016/j.annepidem.2015.03.008 25890796 PMC4599703

[19] Veterans Health Administration. Alcohol use disorder (AUD): leading the charge in the treatment of AUD. Published February 2022. Accessed July 11, 2024. https://www.pbm.va.gov/PBM/AcademicDetailingService/Documents/508/10-1530_AUD_ClinicianGuide_508Conformant.pdf

[20] Wood E, Bright J, Hsu K, ; Canadian Alcohol Use Disorder Guideline Committee. Canadian guideline for the clinical management of high-risk drinking and alcohol use disorder. CMAJ. 2023;195(40):E1364-E1379. doi:10.1503/cmaj.230715 37844924 PMC10581718

[21] Strayer RJ, Friedman BW, Haroz R, . Emergency department management of patients with alcohol intoxication, alcohol withdrawal, and alcohol use disorder: a white paper prepared for the American Academy of Emergency Medicine. J Emerg Med. 2023;64(4):517-540. doi:10.1016/j.jemermed.2023.01.010 36997435

[22] Centers for Disease Control and Prevention. Preexposure prophylaxis for the prevention of HIV infection in the United States—2021 update clinical practice guideline. Published December 2021. Accessed February 20, 2025. https://stacks.cdc.gov/view/cdc/112360

[23] Jophlin LL, Singal AK, Bataller R, . ACG clinical guideline: alcohol-associated liver disease. Am J Gastroenterol. 2024;119(1):30-54. doi:10.14309/ajg.0000000000002572 38174913 PMC11040545

[24] U.S. Department of Veterans Affairs, U.S. Department of Defense. VA/DoD clinical practice guideline for the management of substance use disorders. Published August 2021. Accessed July 11, 2024. https://www.healthquality.va.gov/guidelines/MH/sud/VADoDSUDCPG.pdf

[25] Cohen J, Wilson A, Faden L. Off-label use reimbursement. Food Drug Law J. 2009;64(2):391-403. 19999289

[26] Shuper PA, Joharchi N, Bogoch II, . Alcohol consumption, substance use, and depression in relation to HIV Pre-Exposure Prophylaxis (PrEP) nonadherence among gay, bisexual, and other men-who-have-sex-with-men. BMC Public Health. 2020;20(1):1782. doi:10.1186/s12889-020-09883-z 33256651 PMC7706215

[27] Shuper PA, Varatharajan T, Kinitz DJ, . Perceived influence of alcohol consumption, substance use, and mental health on PrEP adherence and condom use among PrEP-prescribed gay, bisexual, and other men-who-have-sex-with-men: a qualitative investigation. BMC Public Health. 2022;22(1):1875. doi:10.1186/s12889-022-14279-2 36207757 PMC9540691

[28] Substance Abuse and Mental Health Services Administration. 2023 National Survey on Drug Use and Health (NSDUH) releases. Published 2024. Updated February 13, 2025. Accessed March 18, 2025. https://www.samhsa.gov/data/release/2023-national-survey-drug-use-and-health-nsduh-releases

[29] Garcia A, Rowe C, Turner C, Santos GM. Correlates of alcohol-using network size among men who have sex with men in San Francisco, CA. Am J Mens Health. Published online April 24, 2021. doi:10.1177/15579883211007005 33899602 PMC8076769

[30] Hess KL, Chavez PR, Kanny D, DiNenno E, Lansky A, Paz-Bailey G; NHBS Study Group. Binge drinking and risky sexual behavior among HIV-negative and unknown HIV status men who have sex with men, 20 US cities. Drug Alcohol Depend. 2015;147:46-52. doi:10.1016/j.drugalcdep.2014.12.013 25555622 PMC4579526

[31] Johnson M, Jackson R, Guillaume L, Meier P, Goyder E. Barriers and facilitators to implementing screening and brief intervention for alcohol misuse: a systematic review of qualitative evidence. J Public Health (Oxf). 2011;33(3):412-421. doi:10.1093/pubmed/fdq095 21169370

[32] Knox J, Hasin DS, Larson FRR, Kranzler HR. Prevention, screening, and treatment for heavy drinking and alcohol use disorder. Lancet Psychiatry. 2019;6(12):1054-1067. doi:10.1016/S2215-0366(19)30213-5 31630982 PMC6883141

[33] Sharma V, Falise A, Bittencourt L, Zafaranian A, Hai AH, Lopez-Quintero C. Missing opportunities in the screening of alcohol use and problematic use, and the provision of brief advice and treatment information among individuals with alcohol use disorder. J Addict Med. 2024;18(4):408-417. doi:10.1097/ADM.0000000000001301 38587310 PMC11290991

[34] Khan MR, Young KE, Caniglia EC, . Association of alcohol screening scores with adverse mental health conditions and substance use among US adults. JAMA Netw Open. 2020;3(3):e200895. doi:10.1001/jamanetworkopen.2020.0895 32163167 PMC7068229

[35] Hardy C, Fairley CK, Ong JJ, . Drug and alcohol use with condomless anal sex among men who have sex with men in Melbourne, Australia: a retrospective data analysis from 2011 to 2017. Arch Sex Behav. 2022;51(5):2497-2507. doi:10.1007/s10508-021-01966-1 34757603

[36] Candler E, Naeem Khan M, Gratrix J, . Retrospective audit of a convenience cohort of individuals on HIV pre-exposure prophylaxis in Alberta, Canada. J Assoc Med Microbiol Infect Dis Can. 2022;7(4):350-363. doi:10.3138/jammi-2022-0016 37397818 PMC10312220

[37] Curry SJ, Krist AH, Owens DK, ; US Preventive Services Task Force. Screening and behavioral counseling interventions to reduce unhealthy alcohol use in adolescents and adults: US Preventive Services Task Force recommendation statement. JAMA. 2018;320(18):1899-1909. doi:10.1001/jama.2018.16789 30422199

[38] Ghosh A, Morgan N, Calvey T, . Effectiveness of psychosocial interventions for alcohol use disorder: a systematic review and meta-analysis update. Am J Drug Alcohol Abuse. 2024;50(4):442-454. doi:10.1080/00952990.2024.2350056 38904466

[39] Starks TJ, Samrock S, Lopez D, Bradford-Rogers J, Marmo J, Cain D. Testing the effectiveness of a motivational interviewing intervention to reduce HIV risk and drug use in young sexual minority men in a community-based organization setting. AIDS Behav. 2024;28(1):26-42. doi:10.1007/s10461-023-04191-0 37803244 PMC10873079

[40] Karnik NS, Kuhns LM, Hotton AL, . Findings from the Step Up, Test Up study of an electronic screening and brief intervention for alcohol misuse in adolescents and young adults presenting for HIV testing: randomized controlled efficacy trial. JMIR Ment Health. 2023;10:e43653. doi:10.2196/43653 36989027 PMC10131684

[41] Wray TB, Chan PA, Kahler CW, Ocean EMS, Nittas V. Pilot randomized controlled trial of Game Plan for PrEP: a brief, web and text message intervention to help sexual minority men adhere to PrEP and reduce their alcohol use. AIDS Behav. 2024;28(4):1356-1369. doi:10.1007/s10461-023-04223-9 37971613 PMC10947926

[42] Santos GM, Ikeda J, Coffin P, . Targeted oral naltrexone for mild to moderate alcohol use disorder among sexual and gender minority men: a randomized trial. Am J Psychiatry. 2022;179(12):915-926. doi:10.1176/appi.ajp.20220335 36285404 PMC10072332

[43] Carnevale C, Zucker J, Womack JA, . Adolescent preexposure prophylaxis administration: an education curriculum for health care providers. J Pediatr Health Care. 2019;33(3):288-295. doi:10.1016/j.pedhc.2018.09.007 30594441 PMC8459443

[44] Kay ES, Pinto RM. Is insurance a barrier to HIV preexposure prophylaxis? Clarifying the issue. Am J Public Health. 2020;110(1):61-64. doi:10.2105/AJPH.2019.305389 31725314 PMC6893325

[45] Sullivan PS, DuBose SN, Castel AD, . Equity of PrEP uptake by race, ethnicity, sex and region in the United States in the first decade of PrEP: a population-based analysis. Lancet Reg Health Am. 2024;33:100738. doi:10.1016/j.lana.2024.100738 38659491 PMC11041841

