# Supplementary material for: Alcohol Use Disorder Diagnoses Among Individuals Who Take HIV Preexposure Prophylaxis
Source: JAMA Netw Open. 2025 Apr 25;8(4):e257295. doi: 10.1001/jamanetworkopen.2025.7295 (PMC12032559; doi:10.1001/jamanetworkopen.2025.7295)
Supplement: Supplement 1. — eTable 1. National Drug Codes for FDA-Approved PrEP Medications eTable 2. Diagnosis Codes for Viral Diseases Treated With TDF/FTC eTable 3. Diagnosis Codes for Alcohol Use Disorder eTable 4. Diagnosis Codes for Mental Health Conditions eTable 5. Diagnosis Codes for Substance Use Disorders eTable 6. Diagnosis and Service Codes for STI Diagnoses and Testing eTable 7. Diagnosis and Service Codes Used to Identify Psychosocial Therapy eFigure. CONSORT Diagram Showing the Cohort Selection of Individuals Who Use PrEP With and Without AUD Diagnoses eTable 8. Baseline Characteristics of Full Sample [file jamanetwopen-e257295-s001.pdf]

Supplemental Online Content

Avanceña ALV, Okoye G, Yokananth R, Norwood A, Schnarrs PW. Alcohol use disorder among individuals who take HIV preexposure prophylaxis. *JAMA Netw. Open*. 2025; 8(4):e257295. doi:10.1001/jamanetworkopen.2025.7295

eTable 1. National Drug Codes for FDA-Approved PrEP Medications

eTable 2. Diagnosis Codes for Viral Diseases Treated With TDF/FTC

eTable 3. Diagnosis Codes for Alcohol Use Disorder

eTable 4. Diagnosis Codes for Mental Health Conditions

eTable 5. Diagnosis Codes for Substance Use Disorders

eTable 6. Diagnosis and Service Codes for STI Diagnoses and Testing

eTable 7. Diagnosis and Service Codes Used to Identify Psychosocial Therapy

eFigure. CONSORT Diagram Showing the Cohort Selection of Individuals Who Use PrEP With and Without AUD Diagnoses

eTable 8. Baseline Characteristics of Full Sample

This supplemental material has been provided by the authors to give readers additional information about their work.

© 2025 Avanceña ALV et al. *JAMA Network Open*.

eTable 1. National Drug Codes for FDA-approved PrEP medications

Drug	NDC
TDF/FTC (Truvada)	00093760756 00093770456 00378193093 00904717207 16714053401 31722056030 33342010607 35356007003 35356007006 35356007030 42291043930 42385095330 42543071904 50090087000 50090087002 50090087003 50436070101 51407011230 52959096903 54569558800 54569558802 54569558803 54868514100 55045348103 60505420203 61919066902 61958070101 65862035430 66336003203 68071211203 68180028706 68258198303 69097020902 69097074102 69238209503 70710136703 72189015602 72189022702 72189031203 72189040303 76282067730
TAF/FTC (Descovy)	61958200201 61958200202
Cabotegravir (Apretude)	49702026423

FDA, Food and Drug Administration; NDC, National Drug Code; PrEP; preexposure prophylaxis; TDF/FTC, tenofovir disoproxil fumarate/emtricitabine; TAF/FTC, tenofovir alafenamide/emtricitabine.

© 2025 Avanceña ALV et al. *JAMA Network Open*.

eTable 2. Diagnosis codes for viral diseases treated with TDF/FTC

	ICD-9-CM codes	ICD-10-CM codes
HIV	V08<br>079.53<br>042	B20<br>Z21<br>B97.35<br>O98.7
HBV	702.0<br>702.2<br>703.0	B18.1<br>B19.11<br>B16.9<br>B16.2

HBV, hepatitis B virus; HIV, human immunodeficiency virus; IDC-9-CM, *International Classification of Diseases, Ninth Revision, Clinical Modification*; IDC-10-CM, *International Classification of Diseases, Tenth Revision, Clinical Modification*; TDF/FTC, tenofovir disoproxil fumarate/emtricitabine.

© 2025 Avanceña ALV et al. *JAMA Network Open*.

eTable 3. Diagnosis codes for alcohol use disorder

	ICD-9-CM codes	ICD-10-CM codes
Alcohol use disorder	303 Alcohol dependence syndrome<br>303.0 Acute alcoholic intoxication<br>303.9 Other and unspecified alcohol dependence<br>291 Alcohol-induced mental disorders<br>291.0 Alcohol withdrawal delirium<br>291.1 Alcohol-induced persisting amnestic disorder<br>291.2 Alcohol-induced persisting dementia<br>291.3 Alcohol-induced psychotic disorder with hallucinations<br>291.4 Idiosyncratic alcohol intoxication<br>291.5 Alcohol-induced psychotic disorder with delusions<br>291.8 Other specified alcohol-induced mental disorders<br>291.9 Unspecified alcohol-induced mental disorders<br>305.0 Alcohol abuse, unspecified	F10.1 Alcohol abuse<br>F10.10 uncomplicated<br>F10.11 in remission<br>F10.12 Alcohol abuse with intoxication<br>F10.13 Alcohol abuse, with withdrawal<br>F10.14 with alcohol-induced mood disorder<br>F10.15 Alcohol abuse with alcohol-induced psychotic disorder<br>F10.18 Alcohol abuse with other alcohol-induced disorders<br>F10.19 with unspecified alcohol-induced disorder<br>F10.2 Alcohol dependence<br>F10.20 uncomplicated<br>F10.21 in remission<br>F10.22 Alcohol dependence with intoxication<br>F10.23 Alcohol dependence with withdrawal<br>F10.24 with alcohol-induced mood disorder<br>F10.25 Alcohol dependence with alcohol-induced psychotic disorder<br>F10.26 with alcohol-induced persisting amnestic disorder<br>F10.27 with alcohol-induced persisting dementia<br>F10.28 Alcohol dependence with other alcohol-induced disorders<br>F10.29 with unspecified alcohol-induced disorder<br>F10.9 Alcohol use, unspecified<br>F10.90 uncomplicated<br>F10.91 in remission<br>F10.92 Alcohol use, unspecified with intoxication<br>F10.93 Alcohol use, unspecified with withdrawal<br>F10.94 with alcohol-induced mood disorder<br>F10.95 Alcohol use, unspecified with alcohol-induced psychotic disorder<br>F10.96 with alcohol-induced persisting amnestic disorder<br>F10.97 with alcohol-induced persisting dementia<br>F10.98 Alcohol use, unspecified with other alcohol-induced disorders<br>F10.99 with unspecified alcohol-induced disorder

IDC-9-CM, *International Classification of Diseases, Ninth Revision, Clinical Modification*; IDC-10-CM, *International Classification of Diseases, Tenth Revision, Clinical Modification*.

eTable 4. Diagnosis codes for mental health conditions

	**ICD-9-CM codes**	**ICD-10-CM codes**
Depression	296.2 Major depressive disorder single episode<br>296.3 Major depressive disorder recurrent episode<br>300.4 Dysthymic disorder<br>311 Depressive disorder, not elsewhere classified	F32 Depressive episode<br>F33 Major depressive disorder, recurrent<br>F34.1 Dysthymic disorder
Anxiety	300 Anxiety, dissociative and somatoform disorders	F40 Phobic anxiety disorders<br>F41 Other anxiety disorders
Post-traumatic stress disorder (PTSD)	309.81 Acute PTSD	F43.1XX PTSD
Bipolar disorder	296.0 Bipolar I disorder, single manic episode<br>296.1 Manic disorder recurrent episode<br>296.4 Bipolar I disorder, most recent episode (or current) manic<br>296.5 Bipolar I disorder, most recent episode (or current) depressed<br>296.6 Bipolar I disorder, most recent episode (or current) mixed<br>296.7 Bipolar I disorder, most recent episode (or current) unspecified<br>296.8 Other and unspecified bipolar disorders	F30 Manic episode<br>F31 Bipolar disorder<br>F34.0 Cyclothymic disorder

IDC-9-CM, *International Classification of Diseases, Ninth Revision, Clinical Modification*; IDC-10-CM, *International Classification of Diseases, Tenth Revision, Clinical Modification*.

© 2025 Avanceña ALV et al. *JAMA Network Open*.

eTable 5. Diagnosis codes for substance use disorders

	ICD-9-CM codes	ICD-10-CM codes
Opioid use order	305.5 Nondependent opioid abuse<br>304.0 Opioid type dependence	F11 Opioid related disorders
Nicotine dependence	305.1: Tobacco use disorder	F17 Nicotine dependence
Cannabis related disorders	305.2 Nondependent cannabis abuse<br>304.3 Cannabis dependence	F12 Cannabis related disorders
Hallucinogen related disorders	305.3 Nondependent hallucinogen abuse<br>304.5 Hallucinogen dependence	F16 Hallucinogen related disorders
Cocaine related disorders	304.2 Cocaine dependence<br>305.6 Nondependent cocaine abuse	F14 Cocaine related disorders
Sedative, hypnotic, or anxiolytic related disorders	305.4 Nondependent sedative, hypnotic or anxiolytic abuse<br>304.1 Sedative, hypnotic or anxiolytic dependence	F13 Sedative, hypnotic, or anxiolytic related disorders
Stimulant related disorders	969.7 Poisoning by psychostimulants	F15 Other stimulant related disorders
Other drug use disorders	305.8 Nondependent antidepressant type abuse<br>305.9 Nondependent other mixed or unspecified drug abuse<br>304.6 Other specified drug dependence<br>304.7 Combinations of opioid type drug with any other drug dependence<br>304.8 Combinations of drug dependence excluding opioid type drug<br>304.9 Unspecified drug dependence<br>292: Drug-induced mental disorders	F18 Inhalant related disorders<br>F19 Other psychoactive substance related disorders
Opioid overdose	965.00 Poisoning by opium (alkaloids), unspecified<br>965.02 Poisoning by methadone<br>965.09 Poisoning by other opiates and related narcotics<br>E850.1 Accidental poisoning by methadone<br>E850.2 Accidental poisoning by other opiates and related narcotics<br>E850.0 Accidental poisoning by heroin<br>E935.0 Heroin causing adverse effects in therapeutic use<br>E935.1 Methadone causing adverse effects in therapeutic use<br>E935.2 Other opiates and related narcotics causing adverse effects in therapeutic use	T40.0 Poisoning by, adverse effect of and underdosing of opium<br>T40.1 Poisoning by and adverse effect of heroin<br>T40.2 Poisoning by, adverse effect of and underdosing of other opioids<br>T40.3 Poisoning by, adverse effect of and underdosing of methadone<br>T40.4 Poisoning by, adverse effect of and underdosing of other synthetic narcotics<br>T40.6 Poisoning by, adverse effect of and underdosing of other and unspecified narcotics
Amphetamine overdose	305.7 Nondependent amphetamine or related acting sympathomimetic abuse<br>304.4 Amphetamine and other psychostimulant dependence	T43.62 Poisoning by, adverse effect of and underdosing of amphetamines
Cocaine overdose	970.8 Poisoning by other specified central nervous system stimulants	T40.5 Poisoning by, adverse effect of and underdosing of cocaine

IDC-9-CM, *International Classification of Diseases, Ninth Revision, Clinical Modification*; IDC-10-CM, *International Classification of Diseases, Tenth Revision, Clinical Modification*.

© 2025 Avanceña ALV et al. *JAMA Network Open*.

eTable 6. Diagnosis and service codes for STI diagnoses and testing

	ICD-9-CM codes	ICD-10-CM codes	CPT
STI	054.1 Genital herpes<br>091-099 Other STIs<br>070 Viral hepatitis<br>079.4 HPV<br>131 Trichomoniasis<br>614.0 Acute salpingitis and oophoritis<br>614.3 Acute parametritis and pelvic cellulitis<br>614.5 Acute or unspecified pelvic peritonitis, female<br>615.0 Acute inflammatory diseases of uterus, except cervix<br>795.05 Cervical HPV DNA test positive<br>795.09 Other abnormal Papanicolaou smear of cervix and cervical HPV<br>795.15 Vaginal HPV DNA test positive<br>795.19 Other abnormal Papanicolaou smear of vagina and vaginal HPV<br>796.75 Anal HPV DNA test positive<br>V01.6 Contact with or exposure to venereal diseases	A51-A64 Other STIs<br>B15-B19 Viral hepatitis<br>N73 Pelvic inflammatory disease<br>R85.81-R85.82 Anal HPV DNA test positive<br>R87.81-R87.82 HPV DNA test positive from female genital organs<br>Z20.2 Contact with and (suspected) exposure to infections with a predominantly sexual mode of transmission<br>Z20.5 Contact with and (suspected) exposure to viral hepatitis	
STI testing or screening	V65.45 Counseling on other sexually transmitted diseases<br>V73.8 Screening examination for other specified viral and chlamydial diseases<br>V73.9 Screening examination for unspecified viral and chlamydial disease<br>V74.5 Screening examination for venereal disease	Z11.3 encounter for screening for infections with a predominantly sexual mode of transmission<br>Z11.5 Encounter for screening for other viral diseases<br>Z11.8 encounter for screening for other infectious and parasitic diseases	86781, 86780, 86592, 86593, 87164 Syphilis<br>86631, 86632, 87110, 87270, 87320, 87490-87492, 87590–87592, 87810, 87850 Chlamydia and gonorrhea<br>87273, 87274, 87528, 87529, 87530, 86696, 87207 HSV<br>86729 LGV<br>87620, 87621, 87622, 88175 HPV<br>80074, 86704–86707, 87340-87341, 87350, 87515, 87516, 87517 HBV<br>80074, 86803, 86804, 87520–87522 HCV<br>87070, 87808, 87810, 87850, 87660 Trichomoniasis

CPT, Common Procedural Terminology; HBV, hepatitis B virus; HCV; hepatitis C virus; HPV, human papillomavirus; HSV, herpes simplex virus; IDC-9-CM, *International Classification of Diseases, Ninth Revision, Clinical Modification*; IDC-10-CM, *International Classification of Diseases, Tenth Revision, Clinical Modification*; LGV, lymphogranuloma venereum; STI, sexually transmitted infection.

© 2025 Avanceña ALV et al. *JAMA Network Open*.

eTable 7. Diagnosis and service codes used to identify psychosocial therapy

	ICD-procedure, CPT, and HCPCS codes	MarketScan codes
Psychotherapy	90832-90838, 90845, 90847-90849, 90853, 90857, 90865, 90875, 90876, 90880, 90900-90902, 90904, 90906, 90908, 90910, 99510, H0004, H0005, H2019, H2020, H2032, H5010, H5020, H5025, T1006, T1012	PROCGRP=135, 136, 137
Alcoholism counseling	V65.42, Z71.41, G0396, G0397, G0443, H0001, H0050, H0015, H0021, H0030	

CPT, Common Procedural Terminology; HCPCS, Healthcare Common Procedure Coding System; IDC-9-CM, *International Classification of Diseases, Ninth Revision, Clinical Modification*; IDC-10-CM, *International Classification of Diseases, Tenth Revision, Clinical Modification*; PROCGRP, procedure group.

© 2025 Avanceña ALV et al. *JAMA Network Open*.

eFigure. CONSORT diagram showing the cohort selection of PrEP users with and without AUD diagnoses

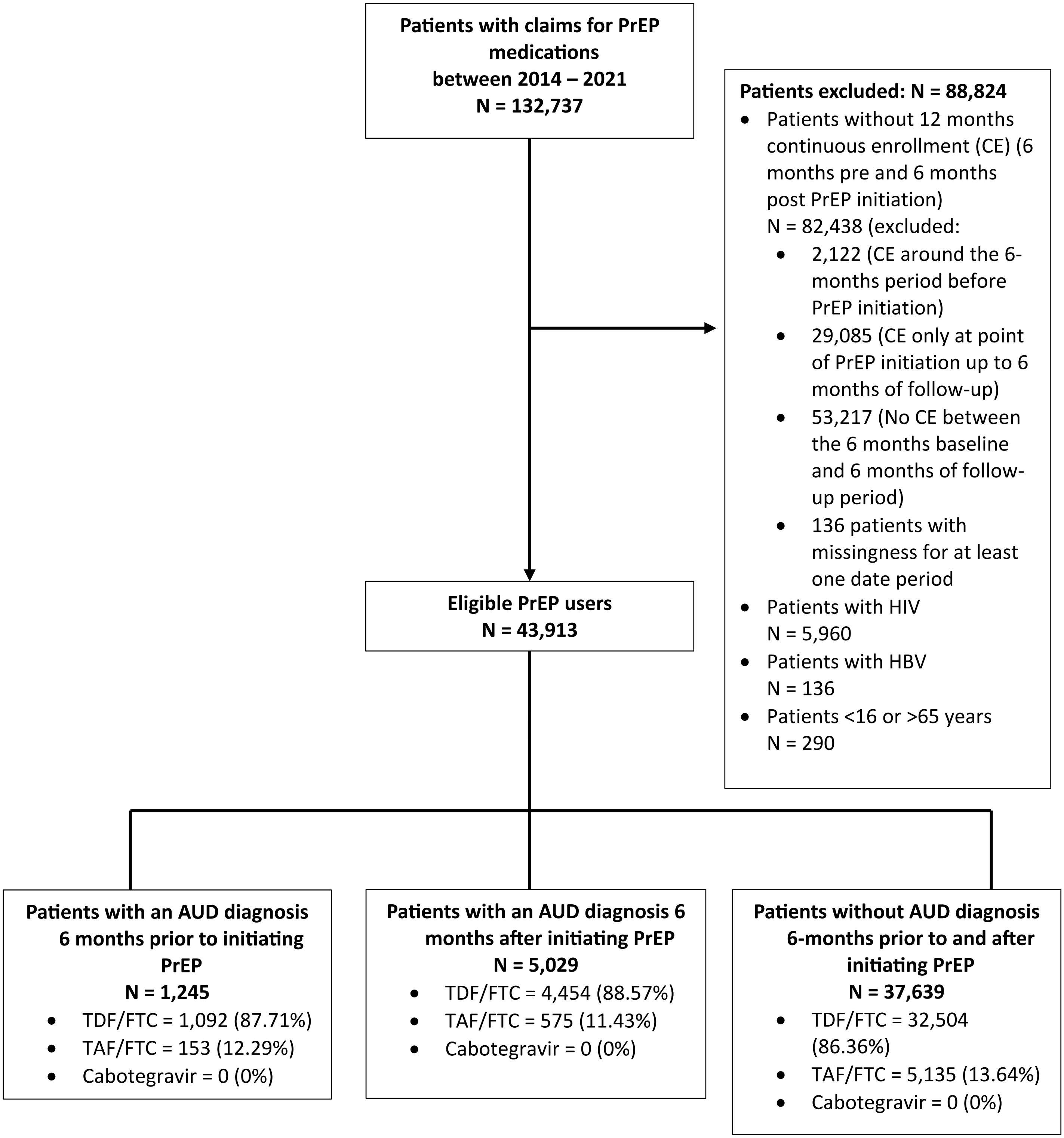

AUD, alcohol use disorder; PrEP; preexposure prophylaxis; TDF/FTC, tenofovir disoproxil fumarate/emtricitabine; TAF/FTC, tenofovir alafenamide/emtricitabine.

© 2025 Avanceña ALV et al. *JAMA Network Open*.

eTable 8. Baseline characteristics of full sample

	**Overall** (N=43,913)
**Age**	
Mean (SD)	35.8 (10.94)
Median	33.0
Range	16.0, 64.0
**Sex at birth**, n (%)	
Female	3857 (9.9%)
Male	35027 (90.1%)
**Index year**, n (%)	
2014-2016	13008 (29.6%)
2017-2019	19330 (44.0%)
2020-2021	11575 (26.4%)
**PrEP medication**, n (%)	
TDF/FTC	38,050 (86.65%)
TAF/FTC	5863 (13.35%)
Cabotegravir	0 (0%)
**Region**, n (%)	
Northeast	9879 (25.5%)
Midwest	5008 (12.9%)
South	14325 (37.0%)
West	9541 (24.6%)
Unknown	131
**Insurance plan**, n (%)	
HMO	5191 (13.3%)
POS	7770 (20.0%)
PPO	17571 (45.2%)
Others	8352 (21.5%)
**Employment status**, n (%)	32395 (83.3%)
**Mental health comorbidities**	
Depression, n (%)	3742 (8.5%)
Anxiety, n (%)	4007 (9.1%)
PTSD, n (%)	1149 (2.6%)
Schizophrenia, n (%)	77 (0.2%)
Bipolar, n (%)	1299 (3.0%)
**Substance use disorders**	
Opioids, n (%)	414 (0.9%)
Nicotine, n (%)	1137 (2.6%)
Cannabis, n (%)	775 (1.8%)
Sedatives, n (%)	297 (0.7%)
Stimulants, n (%)	501 (1.1%)
Other, n (%)	449 (1.0%)
**STI**, n (%)	7129 (16.2%)
**STI screening**, n (%)	4574 (10.4%)
**Psychotherapy**, n (%)	2792 (6.4%)

HMO, health maintenance organization; PrEP, preexposure prophylaxis; POS, point of service; PPO, preferred provider organization; PTSD, posttraumatic stress disorder; SD, standard deviation; STI, sexually transmitted infection; TDF/FTC, tenofovir disoproxil fumarate/emtricitabine; TAF/FTC, tenofovir alafenamide/emtricitabine.

© 2025 Avanceña ALV et al. *JAMA Network Open*.
