# Supplementary material for: Alcohol Use Disorder Diagnoses Among Individuals Who Take HIV Preexposure Prophylaxis
Source: JAMA Netw Open. 2025 Apr 25;8(4):e257295. doi: 10.1001/jamanetworkopen.2025.7295 (PMC12032559; doi:10.1001/jamanetworkopen.2025.7295)
Supplement: Supplement 2. — Data Sharing Statement [file jamanetwopen-e257295-s002.pdf]

Data Sharing Statement

Avanceña. Alcohol Use Disorder Diagnoses Among Individuals Who Take HIV Preexposure Prophylaxis. *JAMA Netw Open*. Published April 25, 2025.
doi:10.1001/jamanetworkopen.2025.7295

Data
Data available: No

Additional Information
Explanation for why data not available: MarketScan® is a licensed database of Merative® and therefore not publicly available. Statistical code is available upon request to the corresponding author (AA). All inquiries should be directed to the corresponding author.
